# Measurement and modeling of polarized specular neutron reflectivity in large magnetic fields

**DOI:** 10.1107/S1600576716007135

**Published:** 2016-06-09

**Authors:** Brian B. Maranville, Brian J. Kirby, Alexander J. Grutter, Paul A. Kienzle, Charles F. Majkrzak, Yaohua Liu, Cindi L. Dennis

**Affiliations:** a NIST Center for Neutron Research, 100 Bureau Drive, Gaithersburg, MD 20899, USA; b Quantum Condensed Matter Division, Oak Ridge National Laboratory, Oak Ridge, TN 37831, USA; c NIST Material Measurement Laboratory, 100 Bureau Drive, Gaithersburg, MD 20899, USA

**Keywords:** polarized neutron reflectometry, applied magnetic fields, Zeeman corrections, non-collinear magnetization

## Abstract

The presence of a large applied magnetic field removes the degeneracy of the vacuum energy states for spin-up and spin-down neutrons. For polarized neutron reflectometry, this must be included in the reference potential energy of the Schrödinger equation that is used to calculate the expected scattering from a magnetic layered structure. For samples with magnetization that is purely parallel or antiparallel to the applied field which defines the quantization axis, there is no mixing of the spin states (no spin-flip scattering) and so this additional potential is constant throughout the scattering region. When there is non-collinear magnetization in the sample, however, there will be significant scattering from one spin state into the other, and the reference potentials will differ between the incoming and outgoing wavefunctions, changing the angle and intensities of the scattering. The theory of the scattering and recommended experimental practices for this type of measurement are presented, as well as an example measurement.

## Abstract 2

A procedure is described for polarized neutron reflectometry when the Zeeman corrections are significant, which occurs when both the magnetic anisotropy and the applied magnetic field are significant. Calculations and a recommended procedure for an example system are provided.

## 1. Introduction   

Polarized specular neutron reflectometry measurements require at least a small magnetic field to be applied throughout the measurement apparatus, in order to maintain a well defined neutron quantization axis. In addition, a larger field is often applied at the sample position in order to manipulate the magnetic state of the sample (Majkrzak *et al.*, 2006 ▸). The difference in the Zeeman energy for a spin-up *versus* a spin-down neutron can lead to observable shifts in both the angle and intensity of scattering for even modest applied fields (tens of millitesla) when spin-flip scattering is appreciable; when the spin-flip cross section is small compared to the non-spin-flip, the corrections remain small.

This so-called Zeeman shift in spin-flipped reflected neutrons was first described by Felcher *et al.* (1995 ▸) and has been observed by many others (Felcher *et al.*, 1996 ▸). Kozhevnikov *et al.* (2012 ▸) presented a clear description of the geometry of the incident and scattered beams. The reflectivity calculation formalism including the Zeeman term was briefly described by van de Kruijs *et al.* (2000 ▸), Fitzsimmon *et al.* (2006 ▸) and Liu *et al.* (2011 ▸), but to our knowledge a detailed description of the calculation is not available in the literature, nor has such a calculation been incorporated into commonly used modeling software.

These shifts are not a major concern in many experiments (Liu *et al.*, 2011 ▸) because the effect is significant only when there is both a large applied field and strong spin-flip scattering. At low fields the corrections are minimal, and at high fields the magnetization tends to align parallel to the applied field, so there is insignificant spin-flip scattering. However, there are important cases where accounting for the Zeeman shift is necessary for appropriately measuring and analyzing data. A technologically relevant example is the study of high-anisotropy magnetic materials used in advanced data-storage applications (Liu *et al.*, 2011 ▸). In such cases the sample magnetization can be non-collinear even with large applied fields.

In this paper we will address the requirements for setting up a measurement in a large field in the case where the spin-flip scattering is not negligible. We present the changes that need to be made to an existing commonly used computer algorithm (implemented in *gepore.f*; Majkrzak *et al.*, 2006 ▸) in order to calculate the scattering correctly, and we present recommended practices for performing the measurements when both the applied magnetic field **H** and the magnetization **M** are large and not parallel to each other. This implies a large magnetic anisotropy in the system. We take advantage of the large shape anisotropy in a thin film of a soft magnetic material in the example experiment section of this paper to show clearly the effects we are discussing.

We must also address the meaning of the word ‘specular’. In many texts on reflectivity the definition is given that the angle of incidence equals the angle of reflection, or that the out-of-plane component of the momentum of the incoming beam *k*_*z*,in_ is equal in magnitude to that of the outgoing reflected beam *k*_*z*,out_. Here we will use a more functional definition based on the momentum transfer **Q** ≡ **k**_in_ − **k**_out_. We define the reflectivity as specular on the condition that the in-plane momentum transfers *Q_x_* = 0 and *Q_y_* = 0, so that the momentum transfer **Q** ≡ 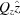 (perpendicular to the surface), as is expected when reflecting from planar layered samples.

As we will demonstrate, some of the kinetic energy along 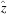 is traded for potential energy during a spin-flip process, so the earlier definitions do not apply in this circumstance, while **Q** remains strictly out of plane.

## 2. Boundary conditions   

Starting with the general Schrödinger equation for a neutron with spin 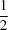,
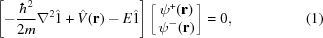
where 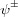 is the spin-dependent wavefunction for the neutron, 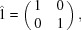


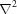
 is the Laplacian (spatial second derivative) and the hatted components indicate a Pauli spin matrix with *z*′ as the quantization axis. We use the notation *z*′ for coordinates in the spin quantization reference frame to distinguish it from the scattering geometric reference frame where *z* is defined to be the surface normal direction for the planar sample, and there is no requirement that 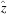 || *z*′. The potential of the particle is made up of a scalar nuclear potential *V*_nuc_ and a magnetic potential due to the field **B**:
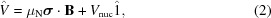
where
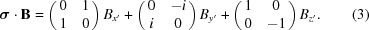
Here, μ_N_ = 5.0507835 × 10^−27^ J T^−1^ is the nuclear magnetic moment.

In the ‘prepared’ spin-polarized beam, we define the direction of the guide field to be 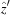, so there are no off-diagonal elements to the potential above (because 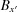 ≡ 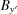 ≡ 0) and the equation decouples into two linear equations for potentials with *V* = *V*_nuc_ ± μ_N_ *B*_*z*′_.

When the beam enters the fronting medium with non-negligible **B**, there is no physical restriction on the direction of **B**, but from an experimental design perspective we note that, if the magnetic field in the fronting medium is not parallel to the applied laboratory field direction 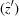, *i.e.* there is a nonzero 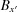 or 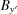 component to the field in this region, the wavefunction will be angularly split owing to the field-dependent difference between 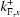 and 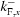. The mutual coherence of the two resulting beams will be impractical to calculate over the macroscopic distances the beam will then travel after being split.

This is not to be confused with the angular splitting which occurs as the beam interacts with the horizontal layers of the sample, which is what is usually being discussed when describing reflectivity, and which is fully taken into account in the calculations below.

**Figure 1:**
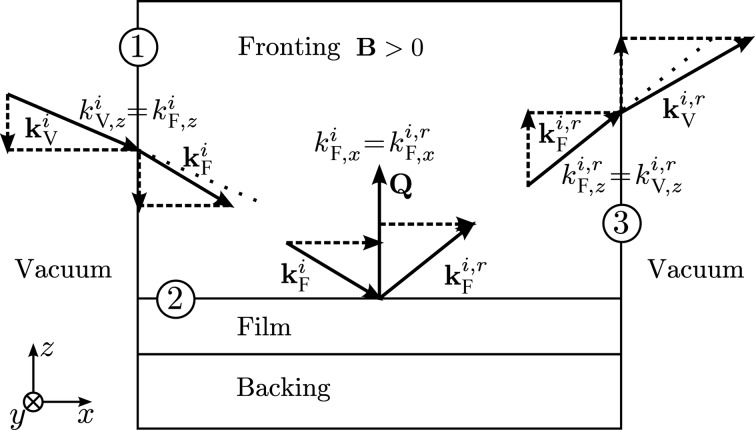
Diagram showing the various components of the **k** and **q** vectors for a polarized specularly reflected neutron entering a magnetic sample system from the left-hand side. The coordinate system is also shown.

Now, restricting ourselves to the case in which the **B** field in the fronting medium is parallel to the guide field outside the fronting medium, we can fully describe the interaction of the neutron with the sample as in Fig. 1 ▸.

The incident neutron is ‘prepared’ in either the *I* ^+^ or *I* ^−^ spin state using techniques described elsewhere (Dura *et al.*, 2006 ▸; Majkrzak *et al.*, 2006 ▸). Neglecting the contribution of a very small magnetic guide field, the total energy of both states is nearly the same for the same **k**_V_ and is equal to the kinetic energy: 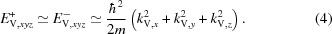
We note that the problem as defined has no *y* dependence: there are no interfaces along that direction (out of the page of the figure) and so the solution for the wave equation along *y* is for a plane wave exp(*ik_y_y*) with constant kinetic energy that can be included in the total energy *E*. The problem can then be treated as a two-dimensional Schrödinger equation in *x* and *z*, with 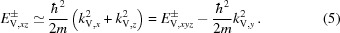
When the neutron enters the fronting medium at the boundary labeled 1 in Fig. 1 ▸, the potential energy changes in a spin-dependent way so that 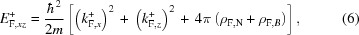


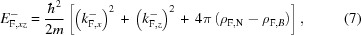
where the notation 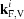 indicates the wavevector in the medium (with the subscripts F and V denoting the fronting and the vacuum, respectively) with spin state *i* (+ or −). The nuclear scattering length density (SLD) of the fronting medium ρ_F,N_ depends on the isotopic composition of the medium, while ρ_F,*B*_ is the magnetic SLD, which can be calculated from the magnetic field in that layer by 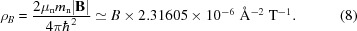
Here μ_n_ and *m*_n_ are the magnetic moment and mass of a neutron, respectively, and *B* is the magnitude of the magnetic field in the fronting medium (in tesla).

Since the magnetic field inside the vertical boundary is parallel to (though possibly much bigger than) the field outside, the (+) or (−) spin state inside the vertical boundary matches the prepared state. Also, by symmetry *k_z_* must be preserved across the vertical boundary labeled 1, between the vacuum and the fronting medium, so *k*_V,*z*_ = *k*_F,*z*_. Since 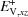 = 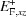 as well, this means that 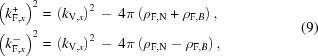
which changes the angle of the neutron beam inside the fronting medium (this is refraction, as indicated by the shortened *k_x_* on the right-hand side of boundary 1 in Fig. 1 ▸). The energy trade in *k_x_* is reversed when the neutron exits the fronting at the boundary labeled 3; the *k*_V,*x*_ on the right is the same as that on the left. This is not in general true for *k*_V,*z*_, as we will see.

Now we consider the next set of boundaries in the problem, namely the horizontal interfaces of the sample under investigation. The first of these is the top interface labeled 2, between the fronting and the sample. When the neutron interacts with this structure, it is possible to have a spin-flip event, so we introduce a second indicator *r* (for reflected) in the notation 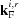 for the spin state of the outgoing neutron (still in the fronting medium). We retain the indicator *i* for the incident neutron spin state because this determines the energy in the fronting, as described above.

We are considering the standard specular reflectometry case where the sample under investigation is homogenous in plane. Thus, while *k_z_* was conserved across boundary 1, now *k_x_* is conserved across boundaries such as 2, so 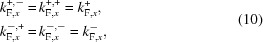
and because the total energy of the neutron is conserved during elastic scattering, we can write 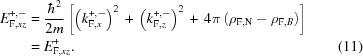
Subtracting this from equation (6) gives 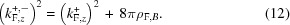
In a similar fashion for the (−) state, we can obtain 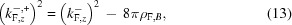
while the non-spin-flipped neutrons are not shifted: 
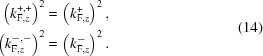
At the next boundary, labeled 3, where the neutrons exit the fronting material and go back into the laboratory environment (vacuum), *k_z_* is again conserved by symmetry, as it was at boundary 1, so the shift in the spin-flipped *k_z_* is carried across this boundary (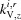 = 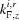 for all *i*, *r*).

The difference between 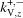 and 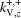 leads to a different propagation direction for the spin-flipped neutron; this measurable angular shift is referred to as Zeeman splitting.

There are values of 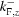 for which 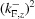 < 8πρ_F,*B*_ and therefore the calculated momentum squared for the spin-flipped reflection 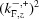 is negative, so that 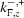 becomes purely imaginary. The calculated amplitude for this reflection is valid at the interface, but this is an evanescent wave that decays as it moves away from the sample. The value of the measured reflectivity corresponds to the amplitude at the detector and is thus effectively zero in this case.

### 2.1. Details of magnetic field geometry   

In the above discussion, the transition from vacuum with zero applied field to a high-field region (also with a possibly nonzero nuclear SLD) was described as a sharp boundary perpendicular to the sample plane (along *x*). In that case, the momentum along *z* is unchanged by the transition, *k*_F,*z*_ = *k*_V,*z*_, and energy conservation leads only to a change in *k_x_*: 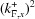 + 4π(ρ_F,N_ + ρ_F,*B*_) = 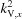.

In real laboratory environments the magnetic field transition is not as abrupt as what is shown in Fig. 1 ▸, and the direction is not perfectly defined, though typically the applied magnetic field is realized in a small volume centered on the sample and the field gradient experienced by the probe neutron is, to first order, radial with respect to the sample. Since for any gradient in the potential the momentum components perpendicular to the gradient direction are conserved throughout the interaction with the potential, the abruptness of the transition is irrelevant and only the direction is important.

So, compared to a more realistic radial magnetic potential gradient parallel to the neutron momentum, we expect that, by using our simplified rectangular boundary conditions (where the sharp gradient at that boundary is along 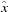 and is nearly but not quite parallel to **k**_in_), we introduce an error in the calculated 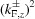 proportional to sin^2^δ, where δ is the angle between the normal to the rectangular boundary and **k**_in_. Because of the right angle between that boundary and the film surface, the result is that δ coincides with the incident angle θ_in_ of the neutron on the film surface.

At the small angles (θ_in_ < 6°) commonly seen for the incident angle during a reflectometry measurement, this results in a maximum correction to 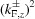 from the model proposed above of the order of 1% of ±4πρ_*B*_ (with the opposite correction made to 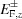). At the even smaller angles (θ_in_ ≃ 0.5°) near the critical edge where these shifts might affect the modeling, the correction is just 0.01% of the magnetic SLD. For this reason, in many cases it is a reasonable approximation that all of the kinetic energy shift in the fronting region prior to the sample is along the *x* direction, as defined by the sample coordinate system in Fig. 1 ▸.

## 3. Calculation of the spin-dependent reflectivity   

### 3.1. One-dimensional Schrödinger equation   

Again considering the region between boundaries 1 and 3 as above, we can calculate the reflectivity of the horizontally layered structure there by reducing the Schrödinger equation to a single spatial dimension *z* and solving with the boundary conditions laid out above. Since the potential is constant as a function of *x* in this region (as it is for *y* everywhere), and *V*(**r**) = *V*(*z*), the one-dimensional spin-dependent Schrödinger equation for the neutron is then (Majkrzak *et al.*, 2006 ▸)
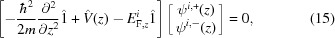
where 
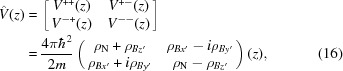
and we fold the constant kinetic energy along *x* into *E* as we did for *y* before:
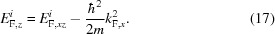


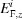
 depends on the spin state of the incident neutron as well as on the potential in the fronting medium, as 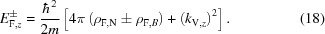
A set of solutions to equation 15 is laid out in the paper by Majkrzak *et al.* (2006 ▸), as (except now keeping track of the polarization *i* of the incident state) 
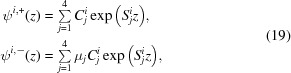
where 
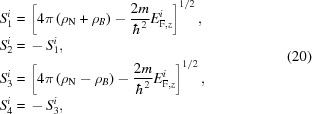
and 
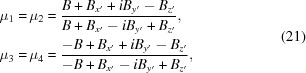
and the terms 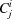 are the complex coefficients of the four components.

Within the fronting medium F the propagation constants *S* are simply equal to the incident wave value *ik*_F,*z*_, since the potentials ρ cancel between equations (18) and (20) for the incident beams *I* ^+^, *I* ^−^.

When the external magnetic potential is negligible, the *E* in the above equations is the same for both incident beam polarizations but, in general, 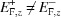 for a sufficiently large field. Because of this, if we measure the reflectivity at the same *k*_V,*z*_ for both the *I* ^+^ and *I* ^−^ states, we have to distinguish between polarization states for the incoming beam.

This distinction based on the Zeeman energy of the neutron in the fronting medium is the basis for a small but critical change to the existing computer code for calculating reflectivity (see *gepore.f*; Majkrzak *et al.*, 2006 ▸). There the term proportional to *E* is set to *Q* ^2^/4 − 4πρ_F,N_ (for *Q* ≡ 2*k*_V,*z*_), which accounts for only the kinetic and nuclear potential energy in the fronting medium; this gives the correct answer for any case except when the Zeeman term is appreciable, so we will use 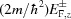 instead, which includes the kinetic, nuclear and magnetic energies in the fronting medium appropriate for the relevant incident spin state.

Also in the previous code, equation (21) for the ratio of ψ^−^ to ψ^+^ components is replaced with
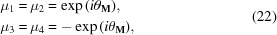
where θ_**M**_ is the in-plane (*x*, *y*) angle, with the underlying implicit assumptions that the contribution to **B** from **H**_applied_ is negligible and that the net *B_z_* (out of the sample plane) is zero. These assumptions are quite reasonable for low values of **H** even when there is a large perpendicular magnetization, because for thin-film samples the demagnetization field |**H**_D_| = *H*_D*z*_ ≃ −*M_z_* almost completely cancels the contribution of the net perpendicular component *M_z_* to *B_z_* [because **B** = μ_0_(**M** + **H**_applied_ + **H**_D_ + ⋯)]. [Of course, the demagnetizing field does not exactly cancel the magnetic field along *z*, and there is a non-zero **B** field at large distances from the sample (measurable with a magnetometer) which is proportional to the volume integral of **M**. In the thin-film geometry, the surface to volume ratio goes to infinity, and this is why there is effectively zero 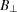 at the surface.]

Now that we are including the effects of an arbitrary external field, however, we must include *B_z_* ≃ *H_z_* and return the more general equation (21) for μ.

Since the applied field along *z* and the associated potential are constant across the sample volume, this does not lead to any additional scattering, which in the continuum limit happens only at discontinuities in the potential. Still, it must be included since it affects (or rather, effects) the relative phase of spin-flipped *versus* non-spin-flipped portions of the neutron wavefunction, which changes the measured reflectivity.

### 3.2. Reparametrization of ψ and reflectivity derivation   

In the more general equation (21), the values of μ_1_ or μ_3_ become unbounded when **B** approaches a direction perfectly parallel or antiparallel to the spin quantization direction 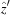. This situation of course always occurs in the fronting (and backing) medium, since there the field direction defines the quantization direction, 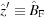. While the equations are analytically correct when one takes the appropriate limits, floating-point computation errors are introduced when multiplying and dividing arbitrarily large numbers in a computer.

Since the μ values in equation (19) serve only to describe the ratio between the components of ψ^+^ and ψ^−^, and because μ_1_ = μ_2_ and μ_3_ = μ_4_, we can rearrange that equation as 
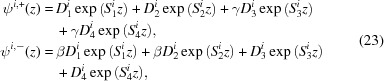
and, relating these constants to our previous parametrization, we obtain 
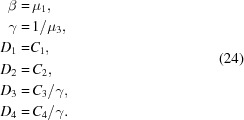
This solution to the Schrödinger equation is valid within any layer of the material, so we can calculate the reflectivity by using the boundary conditions to stitch together solutions from adjacent layers. At any interface, the value of the wavefunction and its first derivative [ψ, ψ′] must be continuous across that boundary. We can write the wavefunction in terms of the 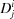 coefficients in that layer (for either incident spin state *i*): 
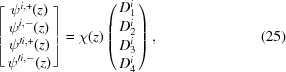
where from equation (23)

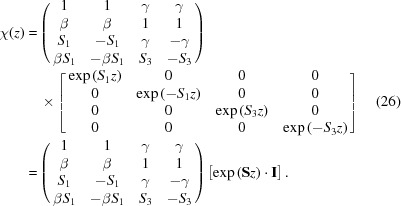
Here γ, β and **S** are specific to the layer *l* and incident spin state *i* being calculated. At the boundary between layers *l* and *l* + 1 (we will define the boundary position *z* ≡ *Z*_*l*_ here), we have ψ_*l*_ = ψ_*l*+1_ and 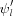 = 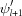, so that 
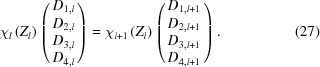
Thus to get {*D*_*l*+1_} from {*D_l_*}, we invert χ_*l*+1_ and 
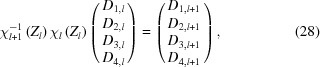
where the formula for χ^−1^ can be calculated to be 
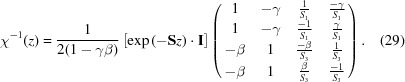
Since γ and β never have the same complex phase, the denominator of equation (29) is never zero. Then for a structure with *N* layers, the coefficients of the transmitted wave 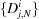 are related to the coefficients in the incident medium 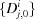 by 
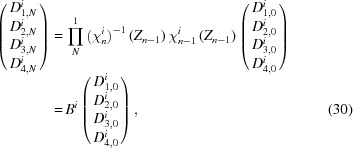
where the pairs of 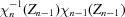 are 4 × 4 matrices. Note that the matrices differ for the different incident spin states, and so we have to calculate the matrix products *B* ^+^ and *B* ^−^ separately. The remaining boundary conditions are met by identifying the coefficients in the fronting medium for the two polarized incident states *I* ^+^ and *I* ^−^, 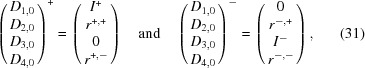
and the coefficients in the backing medium, 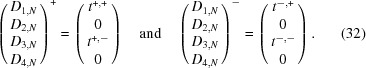
Note that *D*_2,*N*_ and *D*_4,*N*_ are zero because of the boundary condition that the upward-traveling wave coefficient in the backing medium is zero (only downward-traveling waves corresponding to transmission are physical in our experimental setup).

For the *I* ^+^ incident state, *I* ^−^ ≡ 0 and *vice versa*, so we can calculate the ratios *r* ^+,+^ ≡ *r* ^+^/*I* ^+^, *r* ^+,−^ ≡ *r* ^−^/*I* ^+^ *etc.* from the *B* matrix product of equation (30) by using the zeros in *D*_2,*N*_, *D*_4,*N*_, which gives two equations with two unknowns (*r* ^+^, *r* ^−^) if we take the incident intensity to be unity. This gives for the different cross sections 
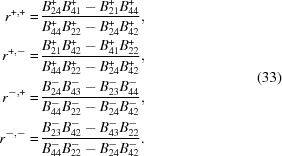
As can been seen in equation (26) above, the new constants γ and β have real physical significance as the mixing terms between ψ^+^ and ψ^−^ in a given layer, and for any *B*_*z*′_ ≥ 0 the constants γ and β are found inside the unit circle in the complex plane, *i.e.* |γ, β| ≤ 1. In the fronting and backing media, they are both identically zero.

For a layer perfectly antiparallel to 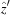, β and γ will still be unbounded, but we further note that the numbering of the roots in equation (20) is arbitrary, so for every layer where *B*_*z*′_ < 0 we perform this switch for the matrix corresponding to that layer: 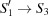, 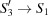, γ′ 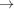 1/β and β′ 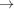 1/γ. The new β′ and γ′ again have a magnitude less than or equal to one, and we can carry on with the calculation. This has no effect on the calculated reflectivity and the matrices are now all well conditioned (the magnitude of the matrix elements is always less than or equal to one). However, if the calculated values of *D_j_* are to be used to reconstruct the full wavefunction within that layer (say, for a distorted-wave Born approximation calculation) one has to be aware of the switch that was made, so that the multiplier *D_j_* is correctly associated with the propagation vector 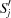 instead of *S_j_*. As in the parallel case, for perfectly antiparallel **B** the mixing terms are exactly zero.

It is interesting that, in this new parametrization, the degenerate case where the magnetization is always aligned parallel or antiparallel to the applied **H** reduces very obviously to two uncoupled equations for the propagation of ψ^+^ and ψ^−^, since the mixing terms in every layer are zero.

Since the spin of the incoming beam is never flipped in this case, the reference energy (including a Zeeman term) for the reflected neutron in the fronting medium will match the energy of the incident neutron for both possible incident spin states, and it can be subtracted from all the equations with no effect as an arbitrary energy offset. Thus, a Zeeman correction to the expected reflectivity will only be needed when there is non-collinear magnetization of the layers, but when this correction has to be made it will alter all the cross sections, including the non-spin-flip reflectivity (because of cross terms in the calculation between spin-flip and non-spin-flip reflectivity).

### 3.3. Parametrization of *k* and *E*   

The wave propagation constants *S* in equation (20) are dependent only on the fixed potentials ρ_*B*_ and ρ_N_ for that layer, and an energy term which depends on the spin state and *k_z_* of the incident neutron. If the reflectivity is solved for a given *E*, this corresponds to a set of 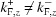: 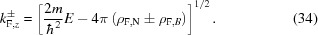
While this saves roughly a factor of two in computation time by mapping a single energy to the corresponding *k* for the two incident spin states, it does not match the way a reflectometry experiment is typically carried out at a reactor-based beamline, where all four spin-dependent cross sections are measured for a single incident wavevector. A more natural instrument coordinate system is based on the incident and reflected angles (θ_in_, θ_out_), which map onto (*k*_in_, *k*_out_), and so we calculate the reflectivity twice for each value of *k*_in_, once for each spin state and the corresponding value of 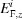.

## 4. Measurement setup   

### 4.1. Sample and detector angles   

While a shift in the reference potential had a large effect on the calculated reflectivities above, it is the angular shift (*i.e.* θ_out_ − θ_in_) in the spin-flipped reflected beam that most affects the instrument setup for this type of measurement.

From the shift in *k_z_* in equations (12) and (13), we can calculate the outgoing angle of the reflected beam by 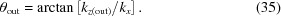
From equation (35), it is easy to see that the angular shift of the spin-flipped reflected beam changes during the measurement, so a position-sensitive neutron detector will clearly facilitate experiments when the Zeeman effect is significant. However, some existing reactor-based polarized neutron reflectivity beamlines use pencil detectors. These have their own advantage of a very high detection efficiency, but an unconventional experimental procedure is required to take care of the Zeeman effect. Below, we detail the experimental setup using a pencil detector when the Zeeman effect is significant. For the four possible spin cross sections, three different values of *k*_*z*(out)_ (and therefore of detector angle) are found for a single *k*_*z*(in)_ in the specular condition [*k*_*x*(in)_ = *k*_*x*(out)_]; one spin-flipped state is shifted higher and the other is shifted lower, while the two non-spin-flip processes give *k*_*z*(in)_ = *k*_*z*(out)_, so that θ_in_ = θ_out_. One could just as well choose a fixed θ_out_ and *k*_*z*(out)_, and calculate the three possible values of *k*_*z*(in)_ for specular scattering, but for this discussion we will use *k*_*z*(in)_ as the fixed quantity.

Since the polarization efficiency of the measurement system typically depends on the instrument geometry, for each of the three θ_out_ corresponding to a specularly reflected beam, all four spin cross sections have to be measured in order to extract an efficiency-corrected reflectivity for that angle. Only one of the corrected reflectivities out of the four will be used from the measurements at Zeeman-shifted angles 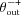 and 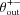, while two reflectivities can be extracted from the non-spin-flip 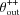 = 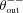 = θ_in_. Overall, this increases the measurement time by a factor of three compared with an experiment without Zeeman corrections.

## 5. Example measurement   

### 5.1. In-plane magnetic sample   

**Figure 2:**
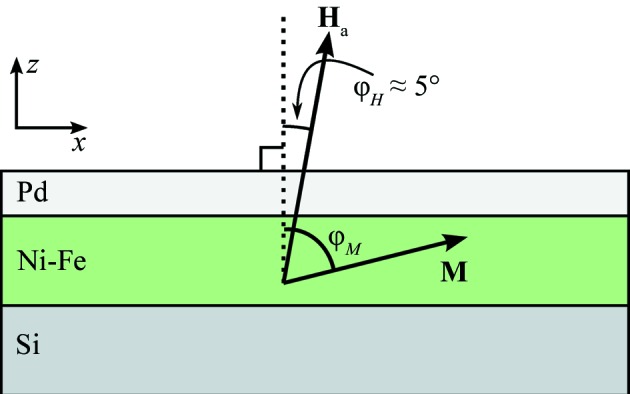
A sketch of the test sample, showing a side view of the layer structure of Pd (200 Å) on an Ni–Fe alloy (600 Å) on an Si substrate. The sample lateral size is 25 × 25 mm. The external applied field is slightly tilted with respect to the surface normal.

In order to realize a large moment non-collinear with the field, a sample of a very magnetically soft material (Ni–Fe alloy) was grown on a single-crystal Si substrate and capped with a layer of Pd to prevent oxidation (as shown in Fig. 2 ▸).

For the principal polarized neutron reflectometry measurement of this study, an external magnetic field was applied for a measurement at a small angle to the film surface normal, as seen in Fig. 2 ▸. The demagnetizing field (shape anisotropy) of the film acts to keep the magnetization in plane, and for appropriate choices of field strength and angle this dominates over the torque from the applied field, so that the magnetization remains largely in plane. At the same time, the small in-plane component of the field *H_x_* is enough to align the layer into a single domain, pointing mostly along *x*.

This arrangement provides an ideal test of the equations, since there is both a large moment **M** 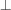 **H** providing spin-flip scattering, and simultaneously a large **H** field which causes Zeeman splitting of the spin-flipped neutrons.

Using a vibrating-sample magnetometer measurement, we verified that the test sample is indeed magnetically soft with a saturation field in the hard (out-of-plane) direction of about 0.5 T, and at 0.244 T (the applied field for the neutron measurements) the out-of-plane loop is linear with field, suggesting a coherent rotation. This verifies that it is a magnetically soft film with the expected shape anisotropy and no significant domain formation under the neutron measurement conditions.

A supplementary reflectometry measurement of the same sample was done in an in-plane saturating field in order to obtain a good value of the saturation magnetization of the soft magnetic layer. The scattering results from this measurement (not shown) are easily fitted to standard models of polarized neutron reflectometry without Zeeman corrections and indicate a saturation internal *B* field of 0.551 T (*M* = 439.53 kA m^−1^). This is below the expected value for an Ni–Fe alloy, which may result from the incorporation of oxygen in the film due to a poor vacuum during the deposition process. Nevertheless, for the purposes of this investigation, all that is required is a magnetically soft film, and the exact magnetization is irrelevant.

### 5.2. Results   

The reflectivity measurements were undertaken at the Polarized Beam Reflectometer instrument (PBR) at the NIST Center for Neutron Research, with a supermirror spin polarizer and analyzer and current-coil Mezei-type spin flippers for the incident and reflected beams. In an applied field μ_0_ *H*_a_ = 244 mT at an angle as shown in Fig. 2 ▸, for a series of *k*_*z*(in)_, all four spin cross sections were measured at each of the three outgoing angles corresponding to 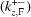, 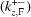 and 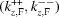. The data for each of those outgoing angles were corrected for polarization and the relevant cross sections were extracted.

**Figure 3:**
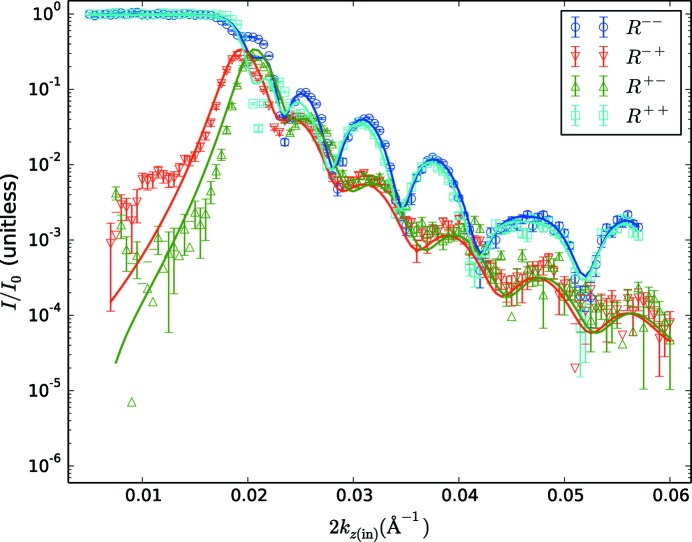
The reflectivity of the test sample and the best fit without including the effects of the Zeeman energy. The data are shown as open symbols, with error bars corresponding to ±1σ according to the counting statistics and the resolution function of the instrument. The fits are shown as solid lines (the reduced χ^2^ for these fits is 25.0). The data were parametrized and fitted according to *k*_*z*(in)_.

In Fig. 3 ▸ we show best fits to the data performed using the freely available *Refl1D* software package (Kirby *et al.*, 2012 ▸; Kienzle *et al.*, 2015 ▸), but without making corrections for the Zeeman effect. The symbols represent data points with error bars, and the lines represent the best fits possible.

**Figure 4:**
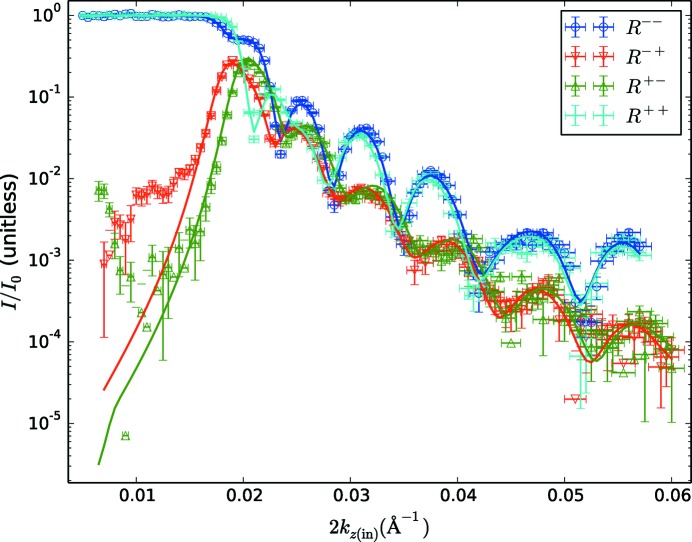
The reflectivity of the test sample, in all four cross sections, including fits. The data are shown as open symbols, with error bars corresponding to ±1σ according to the counting statistics and the resolution function of the instrument. The fits are shown as solid lines (the reduced χ^2^ for these fits is 3.7). The data were parametrized and fitted according to *k*_*z*(in)_.

We compare these with fits performed using a modification of the software, which includes the changes to the theory described in the first part of this manuscript. Both the data and the fits are presented in Fig. 4 ▸.

**Figure 5:**
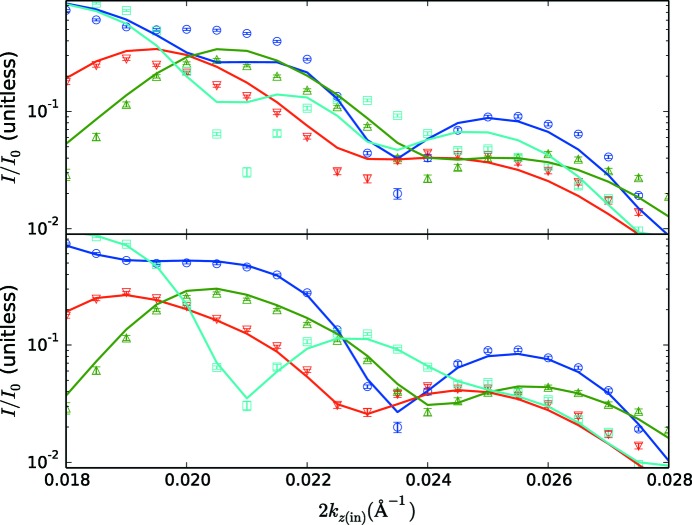
Enlargements of the reflectivity fits near the critical edge. (*a*) Enlargement corresponding to the fits without Zeeman corrections in Fig. 3 ▸. (*b*) Enlargement corresponding to the corrected fits in Fig. 4 ▸. A clear improvement in the quality of the fits is seen. Symbols and lines have the same meanings in this plot as in the originals.

In the uncorrected fits in Fig. 3 ▸, we can clearly see that the splitting between the non-spin-flip scattering at low *k*_*z*(in)_ is grossly underestimated in the best-fitting model. In this region the error bars are small, owing to the strong scattering, and this is what leads to the large minimum χ^2^ value of 25.0 for these fits. An enlargement of this region for comparing corrected *versus* uncorrected fits is shown in Fig. 5 ▸.

By contrast, the Zeeman-corrected fits are very good, with a χ^2^ value of 3.7. The visible deviations of the spin-flip data from the fits at very low *k*_*z*(in)_ are likely to be due to issues with the polarization correction (the correction is of the same magnitude as the spin-flip data there), and this does not significantly affect the rest of the fit. In the enlarged plot in Fig. 5 ▸(*b*), these fits clearly reproduce the data near the critical edge. The best fits to the data correspond to a magnetic SLD in the Ni–Fe layer of ρ_*B*_ = 1.12 × 10^−6^ Å^−2^ and thus *M_x_* = 385 kA m^−1^.

**Figure 6:**
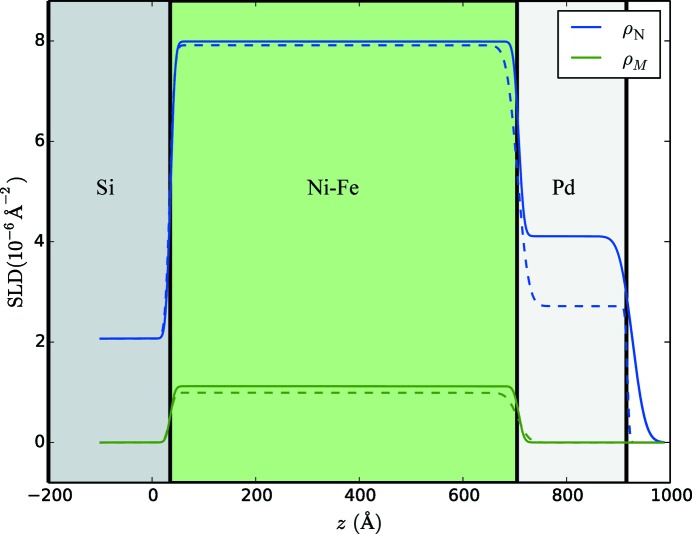
Scattering length density (SLD) profile corresponding to the fits shown in Fig. 4 ▸ (solid line) and Fig. 3 ▸ (dashed line). ρ_N_ and ρ_M_ refer to the nuclear and magnetic SLDs, respectively, in blue and green. For reference, the profiles are overlaid on a color-coded diagram of the sample structure as shown in Fig. 2 ▸.

The SLD profiles (nuclear and magnetic) resulting from the uncorrected fits in Fig. 3 ▸ are shown in Fig. 6 ▸ as dotted lines, while the SLD profiles from the corrected fits in Fig. 4 ▸ are shown as solid lines. The difference between the profiles is most significant in the region of the capping layer, where the uncorrected fit gives an unphysically low value of the nuclear SLD of the Pd capping layer (2.7 × 10^−6^ Å^−2^ rather than the expected value of 4.1 × 10^−6^ Å^−2^), and an unrealistically low roughness for the top interface, which one would expect to have a similar roughness to that of the interface immediately below.

The out-of-plane component of **M** for a system with uniaxial anisotropy arising from the demagnetization field is expected to be linearly dependent (when rotating coherently across the entire sample) on an applied out-of-plane field, reaching its saturation value at *H_k_* = *M*_S_ (where *M*_S_ is the saturation magnetization). In our case, *M_z_* ≃ (*H*_a_/*H_k_*)*M*_S_ = (0.244/0.551)*M*_S_, and since *M_z_* = *M*_S_cosφ and *M_x_* = *M*_S_sinφ, we can extract an expected value for the in-plane magnetization *M_x_* ≃ 394 kA m^−1^, which agrees well with the fitted value of 385 kA m^−1^.

The most striking feature of the scattering in Fig. 4 ▸ is the large splitting between the non-spin-flip reflectivities *R* ^++^ and *R* ^−−^ at low *k*_*z*(in)_, but which disappears at higher *k_z_*. This is a signature of the Zeeman effect, which will be most pronounced when the Zeeman energy is comparable to the kinetic energy along the scattering direction.

The best indication that this splitting is a result of the Zeeman effect is to compare with data fitted to a model with no Zeeman energy included; these are the curves shown in Fig. 3 ▸.

In Figs. 4 ▸ and 3 ▸ there is an apparent horizontal shift between the two spin-flip reflectivities. This is entirely due to the choice of plotting these data as a function of 2*k*_*z*(in)_. If we had chosen to plot the data *versus* the total momentum transfer *Q*, the features would be mostly aligned, but the advantage of plotting the data the way we have is that the scattering sum rules are more apparent; for an incident beam *I* ^−^ at low angles where the scattering is strong, we can clearly see the non-spin-flip reflectivity *R* ^−−^ has a dip when *R* ^−+^ has a peak (a similar correspondence is seen between *R* ^++^ and *R* ^+−^).

## 6. Conclusions   

We have described a procedure for measuring polarized neutron reflectivity in high fields, including important changes to the modeling and instrument configuration due to Zeeman shifts in the energy and angle of spin-flip scattered neutrons. These considerations will be important for the characterization of thin films with a large magnetic anisotropy, which are a component of a growing number of technologically relevant systems.

A data-modeling package with the necessary modifications for this type of measurement has been demonstrated to provide accurate quantitative fits of a test system, and this software is now readily available to the research community (Kienzle, 2015 ▸). The deviations from non-Zeeman-corrected polarized specular neutron modeling are most pronounced where the spin-flip scattering is most intense.
